# The application of multiplex PCR to detect seven different DNA targets in group B streptococci

**DOI:** 10.1007/s12223-012-0108-7

**Published:** 2012-03-13

**Authors:** Tomasz Gosiewski, Monika Brzychczy-Włoch, Piotr B. Heczko

**Affiliations:** Chair of Microbiology, Jagiellonian University Medical College, 18 Czysta Str., 31-121 Cracow, Poland

## Abstract

Group B *Streptococcus* (GBS) causes severe infections in infants and in immunocompromised adults. GBS pathogenicity varies between and within serotypes, with considerable variation in genetic content between strains. For this reason, it is important to be able to carry out immediate and comprehensive diagnostics of these infections. Seven genes important for screening of GBS infection were detected: *cfb* gene encoding the CAMP factor presented in every GBS; the *cps* operon genes such as *cps1aH*, *cps1a*/*2*/*3IJ*, and *cps5O* specific for capsular polysaccharide types Ia, III, and V, respectively; macrolide resistance genes *ermB* and *mefA/E*; and the *gbs2018* S10 region specific for ST17 hypervirulent clone. Standardization of multiplex PCR with the use of seven primer pairs was performed on 81 bacterial strains representing different GBS isolates (*n* = 75) and other Gram-positive cocci (*n* = 10). Multiplex PCR can be used as an effective screening method to detect different sequences important for the screening of GBS infection.

## Introduction

S*treptococcus agalactiae* (group B *Streptococcus*; GBS) is one of the major etiological causes of sepsis and neonatal meningitis in the USA and some European countries (Centers for Disease Control and Prevention 2009; Law et al. 2005). In recent years, GBS has been isolated more often from the immunosuppressed patients (Verani et al. 2010).

Up to the present time, nine serotypes based on the GBS capsular polysaccharides (CPS) have been identified (Ia, Ib, and II–VIII), and as recently a new serotype (IX) has been proposed (Johri et al. 2006; Slotved et al. 2007). In the USA and Europe, serotypes Ia, II, III, and V are found in 80–90% of all clinical isolates (Brimil et al. 2006). On the other hand, serotypes VI and VIII are the most common in Japan (Lachenauer et al. 1999). Polysaccharide capsule has been recognized as one of the most important antiphagocytic virulence factors (Johri et al. 2006). As a result, GBS displays significant differences in virulence depending on the CPS. Serotype Ia is responsible for most cases of early onset disease in newborns, serotype III for late onset disease (Persson et al. 2004). Serotype V, particularly macrolide-resistant strains, is responsible for infections in the elderly or immunodeficient patients (Persson et al. 2004).

In the recent years, the hypervirulent ST17 clone defined with the application of multilocus sequence typing (MLST) method associated with a very high mortality rate in newborns with early onset disease has been of great interest (Manning et al. 2009; Poyart et al. 2008).

Penicillin is the recommended drug because GBS resistance has not been reported. However, for people allergic to penicillin, the alternative drugs are macrolides or lincosamides (Kotarski et al. 2008; Verani et al. 2010). Two main antibiotic resistance mechanisms are described for *S. agalactiae*. The first one is MLS_B_ phenotype characterized by cross-resistance to macrolides, lincosamides, and streptogramins B which are encoded by the *erm* genes (erythromycin ribosomal methylase). The second mechanism is M phenotype which is associated with the expression of the *mefA/E* genes (macrolide resistance) (Gherardi et al. 2007).

As a result, the objective of our studies was to design and standardize the multiplex PCR method as a technique to identify seven different DNA factors of GBS strains: (1) *S. agalactiae* species, (2) serotype Ia, (3) serotype III, (4) serotype V, (5) the macrolide resistance gene *ermB*, (6) the macrolide resistance genes *mef A/E*, and (7) hypervirulent ST17 clone associated with the pathogenicity of GBS whose presence may influence the prescribed therapeutic decisions.

## Materials and methods

### Examined GBS strains

The *S. agalactiae* strains used in the examinations were derived from the collection of the Chair of Microbiology, Jagiellonian University Medical College described in our previous paper (Brzychczy-Włoch et al. 2012). The total of 75 isolates coming from the clinical materials representing seven most important genetic factors significant for GBS diagnostic and epidemiological purposes were selected for this study. The reference GBS strains, such as: 11360 National Collection of Type Cultures (NCTC)—serotype Ia; 2134 DSM (German Collection of Microorganisms and Cell Cultures)—serotype II; 12403 American Type Culture Collection (ATCC)—serotype III; and BAA-611 ATCC—serotype V were tested. The hypervirulent ST17 clone of GBS came from our collection and it was confirmed by MLST method. Moreover, non-GBS strains belonging to the beta-hemolytic Gram-positive cocci that can be most frequently confused with GBS were studied: *Streptococccus pyogenes*—strains 2071 DSM and 20565 DSM, *Enterococcus faecalis*—strains 20478 DSM and 19434 ATCC, *Staphylococcus aureus*—24167 DSM and *Staphylococcus haemolyticus*—strain 29970 ATCC.

### The PCR method to confirm *S. agalactiae* species and detect the examined infection-related factors

For the purpose of DNA isolation the specific bacterial isolates were cultured in the TSB medium (Difco) at the temperature of 37°C for 24 h. The bacterial DNA was isolated with the use of the nucleic acid isolation kit (Genomic Mini, DNA Gdansk) pursuant to the manufacturer’s protocol. Afterwards, the separate PCR amplifications were conducted on the DNA isolates to detect the presence of *cfb* gene encoding the CAMP factor presented in every GBS (Ke et al. 2000); the *cps* operon genes *cps1aH, cps1a*/*2*/*3IJ* and *cps5O* specific for CPS types Ia, III and V, respectively (Poyart et al. 2007); macrolide resistance genes *ermB* and *mefA/E* (Sutcliffe et al. 1996); the *gbs2018* S10 region specific for ST17 hypervirulent clone (Lamy et al. 2006). The detection of ST17 clone was additionally confirmed with the application of the MLST method list of housekeeping genes (Jones et al. 2003) and primers (Manning et al. 2008).

### The multiplex PCR method standardization

The standardization of the method was carried out with the use GBS strains where the presence of at least one out of seven different DNA factors of GBS was confirmed before with the application of monoplex PCR (Table 1). Optimization of the multiplex PCR method was based on the selection of the appropriate concentration of magnesium ion (0.625–5.625 mmol/L), primer (0.1–1.0 mmol/L) as well as determining the appropriate temperature (46–60°C) for all the seven pairs of primers to anneal to the DNA matrix.

**Table 1:** Primers, PCR products specific for the studied gene targets, and conditions for the multiplex PCR reaction

Studied targets	Primer sequences 5′–3′	*T*_m_ [°C]^a^	Primers		PCR products [bp]
			Names	[μmol/L]^b^	
*S. agalactiae*	TTT CAC CAG CTG TAT TAG AAG TA	55.3	Sag59	0.125	153
	GTT CCC TGA ACA TTA TCT TTG AT	55.3	Sag190		
	(Ke et al. 2000)				
Ia serotype	GG TCA GAC TGG ATT AAT GGT ATG C	64.0	Ia-F	0.25	521
	GTA GAA ATA GCC TAT ATA CGT TGA ATG C	64.0	Ia-R		1,826
	(Poyart et al. 2007)				
III serotype	TCC GTA CTA CAA CAG ACT CAT CC	63.0	III-F	0.5	1,826
	AGT AAC CGT CCA TAC ATT CTA TAA GC	63.0	III-R		
	(Poyart et al. 2007)				
V serotype	GAG GCC AAT CAG TTG CAC GTA A	64.0	V-F	0.375	701
	AAC CTT CTC CTT CAC ACT AAT CCT	62.0	V-R		
	(Poyart et al. 2007)				
*mefA/E* gene	AGT ATC ATT AAT CAC TAG TGC	46.5	mefA	0.25	348
	TTC TTC TGG TAC TAA AAG TGG	48.5	mefE		
	(Sutcliffe et al. 1996)				
*erm B* gene	GGA AAG GTA CTC AAC CAA ATA A	46.5	ermB1	0.375	639
	CAT TTG TTA AAT TCA TGG CAA TGA	48.8	ermB2		
	(Sutcliffe et al. 1996)				
ST17 clone	ATA CAA ATT CTG CTG ACT ACC G	51.1	ST17S	0.125	210
	TTA AAT CCT TCC TGA CCA TTC C	51.1	ST17AS		
	(Lamy et al. 2006)				

^a^ *T*_m_ temperature specified by the manufacturer

^b^Concentration of primers in the multiplex reaction mixture

The PCR products were separated after amplification in 2% agarose gel in 0.5 × TBE buffer (Fluka) in the presence of ethidium bromide (0.25 μg/mL) (Sigma). The final analysis was conducted with the application of Quantity One software (BioRad) and gel visualization apparatus GelDoc2000 (BioRad).

After determining the optimal conditions for amplification, the method was tested to check its efficacy. For this purpose, the analysis of selected reference strains possessing the sought genetic markers was performed. Afterwards, the examination of 75 GBS isolates which possessed at least one out of the seven sought DNA sequences and which had previously been subjected to the genetic analysis with the use of separate PCR methods described by other authors (Table 1) was blindly performed. Moreover, six non-GBS strains belonging to the beta-hemolytic Gram-positive cocci that can be most frequently confused with GBS were tested: *Streptococcus pyogenes*, *Enterococcus faecalis*, *Streptococcus aureus*, and *Streptococcus haemolyticus*. Additionally, the multiplex PCR reaction was carried out using the DNA isolated from the genital tract swabs of women colonized with GBS in order to check the efficacy of the designed method in such a case.

## Results

The optimal conditions for conducting the amplification of *S. agalactiae* DNA were determined for simultaneous detection of seven most frequent genetic factors significant for diagnostic and epidemiological purposes. The annealing temperature of the primers was determined as ramp 0.3°C/s up to 52.0°C. The optimal concentration of specific pairs of primers was determined in an experimental way and presented in Table 1. The primer concentration was decreased for shorter products, whereas it was increased for the longer ones.

**Fig. 1:**
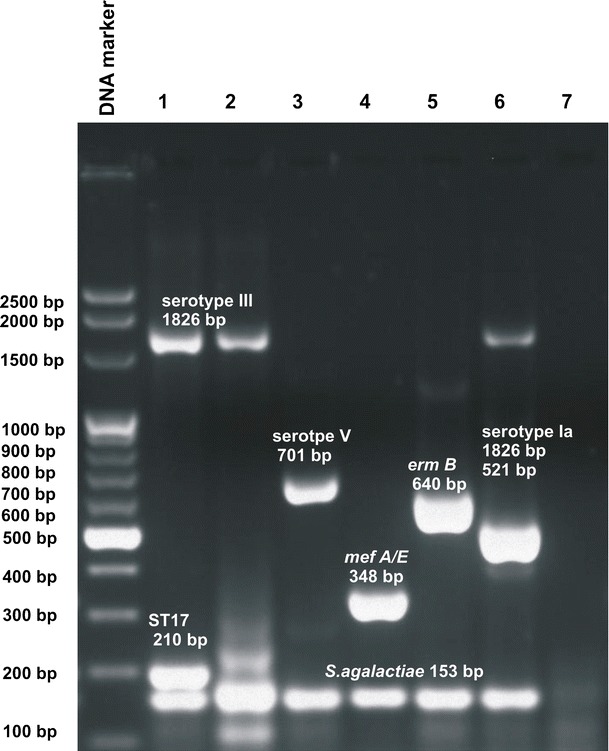
Results of standardization of the multiplex PCR for detection of seven different sequences important for screening of group B *Streptococcus* infection (*1*—hypervirulent clone ST17; *2*—12403 ATCC, III serotype; *3*—BAA611 ATCC, V serotype; *4*—strain with *mef A/E* gene; *5*—strain with *ermB* gene; *6*—11360 NCTC; Ia serotype; *7*—the negative control)

The total volume of the designed multiplex PCR reaction was 40 μL and it consisted of: seven pairs of primers (concentration presented in Table 1); 50 ng of bacterial DNA; 250 μmol/L of each dNTP (Fermentas); 1.87 mmol/L MgCl_2_, 2 U of *Taq* polymerase (EURx); and KCl buffet for polymerase (EURx). The developed program for multiplex PCR amplification was devised using the following thermal profile: 95°C—5 min, 50 × (95°C—1 min, ramp 0.3°C to 52°C, 52°C—1 min, 72°C—3 min), 72°C—10 min (PTC-200, BioRad). The efficacy of the multiplex PCR method was tested with the application of the group of reference strains with confirmed presence of the desired genetic sequences (Fig. 1).

Compatibility with the results obtained from separate PCR reactions in the monoplex system was 100%. Furthermore, it was proved that designed multiplex PCR method applied in DNA samples derived from other Gram-positive bacteria, such as *S. pyogenes*, *E. faecalis*, *S. aureus*, and *S. haemolyticus*, did not provide a positive signal after gel electrophoresis which meant that it was characteristic only for *S. agalactiae* bacteria. Sensitivity of our method was too low to detect the above-mentioned sequences in DNA samples isolated directly from the genital tract swabs.

## Discussion

Infections triggered off by *S. agalactiae* are the problems particularly in newborns which often lead to death due to occurrence of sepsis or meningitis (Verani et al. 2010). In recent years, GBS infections among the elderly or the immunosuppressed patients have been more frequently observed (Thigpen et al. 2011). The classical microbiological diagnosis of these infections is most frequently reduced to bacterial culture and assessment of their drug resistance, which is time-consuming and lowers the chances for effective treatment. Due to this reason, other diagnostic methods which will allow simultaneously accurate detection of GBS presence as well as defining their most important determinants, such as resistance to the most common antibiotics, serotypes, or affinity to hypervirulent ST17 clone, are essential. Actually, this can only be achieved by molecular biology methods which are more specific than methods based on phenotypic analysis of GBS.

The multiplex PCR method described in this study allows the simultaneous detection of as many as seven different DNA targets, which can both confirm the presence of *S. agalactiae* bacteria as well as depict the most common serotypes in Europe and the USA (Ia, III, and V), macrolide resistance genes (*ermB* and *mefA/E*), and affinity to the hypervirulent ST17 clone (Brimil et al. 2006; Gherardi et al. 2007; Manning et al. 2009; Poyart et al. 2008). This gives our method a significant predominance over the commercial tests since it provides important data as far as clinical and epidemiological points of view are concerned. In our research in Poland, serotype III was predominant (35%), then serotype Ia (20%) and serotype V (17%) constituting the total of 72% (Brzychczy-Włoch et al. 2012). The *ermB* gene was indicated in 63% of all the isolates with cMLSB phenotype and serotype V, whereas the *mefA/E* genes were found in 11% of all the isolates (Brzychczy-Włoch et al. 2010; Pinheiro et al. 2009).

The PCR method is very sensitive and theoretically it allows to detect single copies of DNA sequences in the analyzed sample. However, the simultaneous amplification of several different DNA sequences during the single PCR reaction decreases the reaction sensitivity. In the research, this resulted in the lack of amplification signal while conducting the PCR in DNA samples isolated directly from the material from the genital tract. Furthermore, DNA amplification is sensitive to numerous inhibitors which can be included in the DNA isolate from the clinical material (Domeika and Drulyte 2000; Lefevre et al. 2003). In addition, isolation of nucleic acids from such a material results in the presence of a significant amount of human DNA sequences which may interact with bacterial DNA, which evidently lowers PCR sensitivity (Cogswell et al. 1996).

Designing the multiplex PCR method used to detect the seven DNA targets in the single reaction of amplification constitutes a remarkable achievement considering the fact that it was possible to combine the starters coming from independent scientific publications as well as develop one joint amplification program in order to obtain considerably different-sized products of the PCR reaction (153–1,826 bp). What is more, this method can constitute a perfect tool for diagnostic and epidemiological screening in hospitals with the access to diagnostic laboratories equipped with the basic equipment for molecular research. Due to its application, it is possible to confirm the GBS presence as well as detect the macrolide resistance genes, the hypervirulent ST17 clones, and the most common serotypes Ia, III, and V related to high morbidity and mortality rates in patients.

This multiplex PCR can be used as a screening method to detect the most frequent determinants of GBS infections. We also hope that the described method will allow the more precise testing of cases of suspected GBS infection.
